# Clinical and pathological features of second primary neoplasms arising in head and neck reconstructive skin flaps

**DOI:** 10.1038/s41598-023-38122-9

**Published:** 2023-07-11

**Authors:** Kohtaro Eguchi, Kenya Kobayashi, Yoshitaka Honma, Eijitsu Ryo, Airi Sakyo, Kazuki Yokoyama, Takane Watanabe, Yusuke Aihara, Azusa Sakai, Yoshifumi Matsumoto, Toshihiko Sakai, Go Omura, Yasushi Yatabe, Seiichi Yoshimoto, Taisuke Mori

**Affiliations:** 1 Department of Head and Neck Surgery, National Cancer Center Hospital, 5-1-1, Chuo-ku, Tokyo, 104-0045 Japan; 2 Department of Otolaryngology, Head and Neck Surgery, The University of Tokyo, 7-3-1, Hongo, Bunkyo-ku, Tokyo, 113-8655 Japan; 3 Department of Head and Neck, Esophageal Medical Oncology, National Cancer Center Hospital, 5-1-1, Chuo-ku, Tokyo, 104-0045 Japan; 4 Department of Diagnostic Pathology, National Cancer Center Hospital, 5-1-1, Chuo-ku, Tokyo, 104-0045 Japan; 5 Division of Molecular Pathology, National Cancer Center Research Institute, 5-1-1, Chuo-ku, Tokyo, 104-0045 Japan

**Keywords:** Head and neck cancer, Oncogenes, Oral cancer

## Abstract

The incidence of second primary neoplasms arising in the skin reconstructive flap (SNAF) is increasing because of the increase in head and neck flap reconstruction and cancer survival. Prognosis, optimal treatment, and their clinicopathological-genetic features are under debate and are difficult to diagnose. We retrospectively reviewed SNAFs based on a single center’s experience over 20 years. Medical records and specimens of 21 patients with SNAF who underwent biopsies between April 2000 and April 2020 at our institute were retrospectively analyzed. Definite squamous cell carcinoma and the remaining neoplastic lesions were subclassified as flap cancer (FC) and precancerous lesions (PLs), respectively. Immunohistochemical studies focused on p53 and p16. *TP*53 sequencing was conducted using next-generation sequencing. Seven and 14 patients had definite FC and PL, respectively. The mean number of biopsies/latency intervals was 2.0 times/114 months and 2.5 times/108 months for FC and PL, respectively. All lesions were grossly exophytic and accompanied by inflamed stroma. In FC and PL, the incidences of altered p53 types were 43% and 29%, respectively, and those of positive p16 stains were 57% and 64%, respectively. Mutation of *TP*53 in FC and PL were 17% and 29%, respectively. All except one patient with FC under long-term immunosuppressive therapy survived in this study. SNAFs are grossly exophytic tumors with an inflammatory background and show a relatively low altered p53 and *TP*53 rate and a high p16 positivity rate. They are slow-growing neoplasms with good prognoses. Diagnosis is often difficult; therefore, repeated or excisional biopsy of the lesion may be desirable.

## Introduction

Despite the surprising pathological variety, surgery plays a significant role in treating head and neck tumors. Since the introduction of flap reconstruction by Bakamjian et al.^1^, radical resection has pushed back the boundaries of previous decades, reinforced by evolving flap reconstruction techniques. Meanwhile, remarkable refinements in chemotherapy and radiotherapy and the phenomenal introduction of immune checkpoint therapy have enabled the long-term survival of patients with cancer. In this new era, second primary carcinomas arising in the skin paddle of the flap, known as flap cancer, have been reported (Supplementary Material 1)^2–21^. This carcinogenesis is relatively unique; therefore, the evidence is limited to a few sporadically reported cases. In addition, the etiology of the disease is under study, and the definition of flap cancer remains ambiguous. Furthermore, differentiating between flap cancers and local relapse of the primary disease is occasionally difficult, although the treatment may differ. Therefore, to overcome these issues, this study aimed to elucidate the etiologic features of flap cancers through clinical and pathological evaluation of cases accumulated over 20 years, with the largest cohort in a single report. To better understand the disease’s etiology, the medical term “second primary neoplasm arising in the skin paddle of the reconstructive flap (SNAF)” is used in this study.

## Methods

### Patients and lesions

This was a retrospective study using data from our institutional medical records and pathological archives. SNAF was suspected when a grossly abnormal lesion was spatially related to the reconstructed skin paddle. Biopsies were performed on 26 Japanese patients with suspected SNAF at our institute between April 2000 and April 2020. In this study, lesions fulfilling the following three criteria were defined as local relapses and excluded from the study: (1) the lesion was identified within 5 years of the last therapy, (2) the initial surgical margin was positive for the tumor, and (3) the lesion was located at the periphery of the skin paddle. Five cases defined as local relapses were excluded, and a total of 21 patients were retrospectively evaluated. Furthermore, the latency interval was defined as the period between the completion of the initial therapy and the final biopsy, which confirmed the diagnosis of SNAF.

To elucidate the clinical features of SNAF compared with those of typical head and neck squamous cell carcinoma (HNSCC), the data of patients with HNSCC who were initially surgically treated at our institute between January 2013 and December 2015 were reviewed as a control cohort. Patients with the following features were excluded from this control cohort: p16-positive oropharyngeal squamous cell carcinoma; second primary tumor; primary tumor of the nasopharynx, external ear canal, or salivary gland; and cervical metastasis of an unknown primary tumor. Detailed clinical and genomic information of the patients and the primary tumor are described in our previous reports^22–24^.

All of the patients provided written informed consent and the study was approved by the institutional revies boards of the National Cancer Center Hospital, Japan (Approval No. 2018-179). The study was conducted following the Declaration of Helsinki and Ethical Guidelines for Medical Research Concerning Humans of the Ministry of Education, Culture, Sports, Science, and Technology, Japan.

### Pathology

Pathological information was obtained and reviewed from the initial surgical specimen to the final biopsy specimen for the 21 patients enrolled in this study. Furthermore, longitudinal pathological changes in biopsy specimens were assessed, and the distinction between them and the initial surgical specimens was clarified.

Immunohistochemical (IHC) studies focused on p53 (Dako Omnis, clone DO-7) and p16 (Ventana, clone E6H4). For p53 expression, IHC results were classified into wild-type and altered types. Non-regionalized patchy stains were judged as “ +/−”; these lesions were categorized as wild-type. In addition, lesions lacking stain were considered as “−” (lost), weak or equivoval stain were considered as “ + ” and strong regionalized stains were judged as “ ++ ” (accumulation); these were classified as the altered type (Supplementary Material l 2). Lesions with significantly increased p16 expression in both the nuclei and cytoplasm in at least 70% of the neoplastic cells were defined as p16-positive tumors.

Among the 21 lesions biopsied with suspicion of SNAF based on macroscopic findings, those with definite evidence of malignancy were classified as flap cancers. Moreover, among the SNAFs, those with dysplasia and severe cellular and structural atypia were pathologically diagnosed as precancerous lesions.

### TP53 sequencing

DNA extraction and sequential analysis were performed using our stocked FFPE biopsy specimens. Paraffin blocks of specimens were deparaffinized, microdissected, and subjected to DNA extraction using a Gene-Read DNA FFPE Tissue Kit (Qiagen, Hilden, Germany). Next-generation sequencing (NGS) libraries were prepared via two-step tailed PCR; each pool contained 36 pairs of primers targeting frequently mutated regions of *TP*53. Pair-stich read sequencing was performed using MiSeq (Illumina,San Diego, CA, USA) and MiSeq Reagent Micro Kit v2 (300 cycles) (Illumina). Sequence variations with variant allele frequencies and depths of more than 5% and 100×, respectively, were identified as candidate mutations. More detailed sequencing protocols are available in our previous reports^22,23^.

### Statistical analysis

Differences in the distribution of baseline characteristics between the control cohort, patients with flap cancer, and those with precancerous lesions were evaluated using Fisher’s exact test. In addition, the Kaplan–Meier survival method was used to estimate the median disease-specific survival. Survival time was defined as the number of survival months from the date of the last biopsy until the date of death or the last visit for the SNAF group and the completion of initial therapy until the date of death or the last visit for the control cohort. Finally, we tested for crude differences between the SNAF and control cohorts using a log-rank test. All statistical tests were two-sided, and 95% confidence intervals were calculated. Furthermore, all statistical analyses were conducted using EZR (Saitama Medical Center, Jichi Medical University, Japan), a graphical user interface for R (The R Foundation for Statistical Computing, Vienna, Austria).

## Results

### Flap cancer

**Figure 1:**
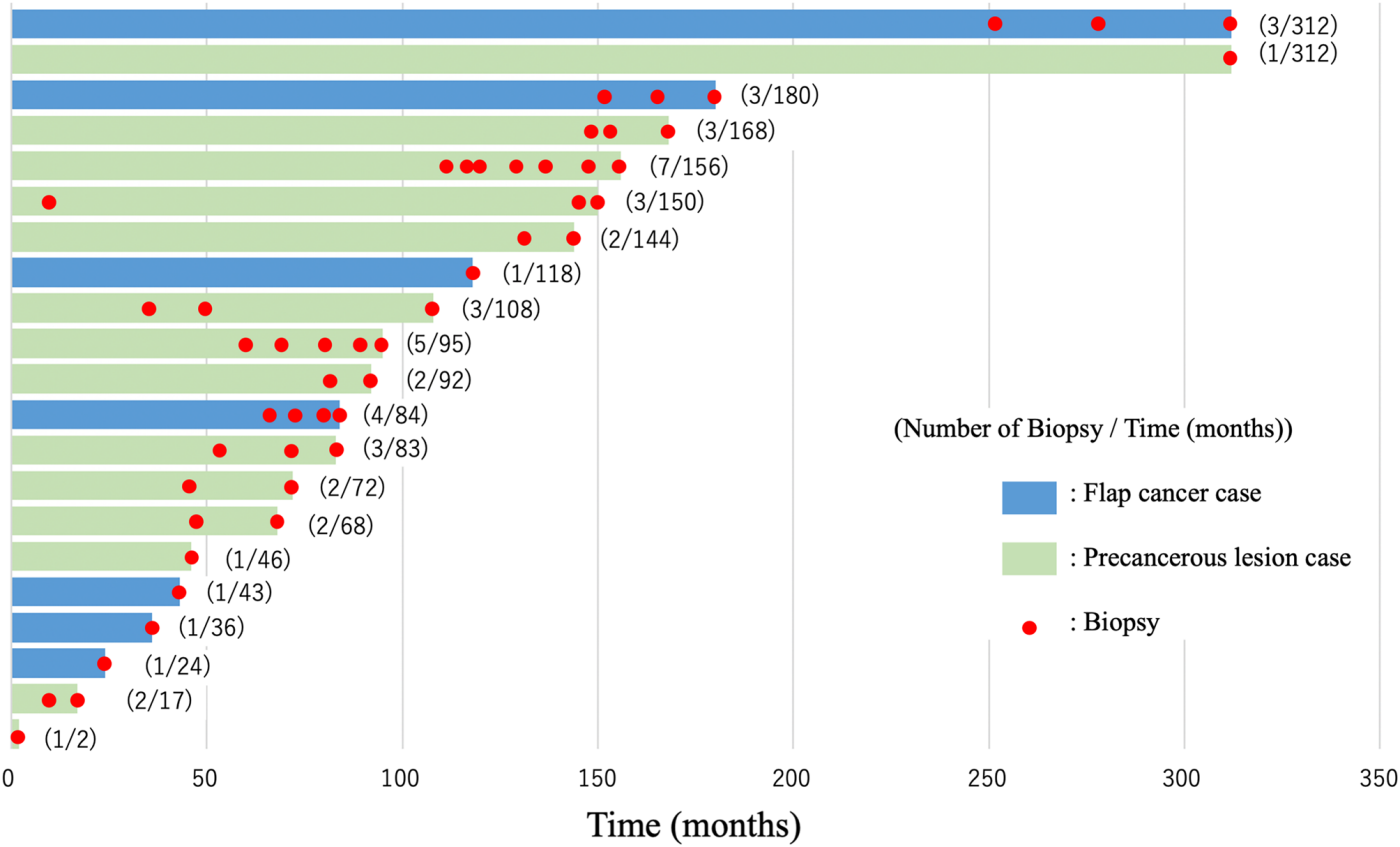
Number of biopsies and latency intervals of all cases. Time is the latency interval and the number of months from the completion of the initial therapy until the date of the last biopsy. The mean number of biopsies/latency intervals were 2.0 times/114 months and 2.5 times/108 months for flap cancer and precancerous lesions, respectively. Diagnosing flap cancer and precancerous lesions tended to require repeated biopsies over a long period.

**Figure 2:**
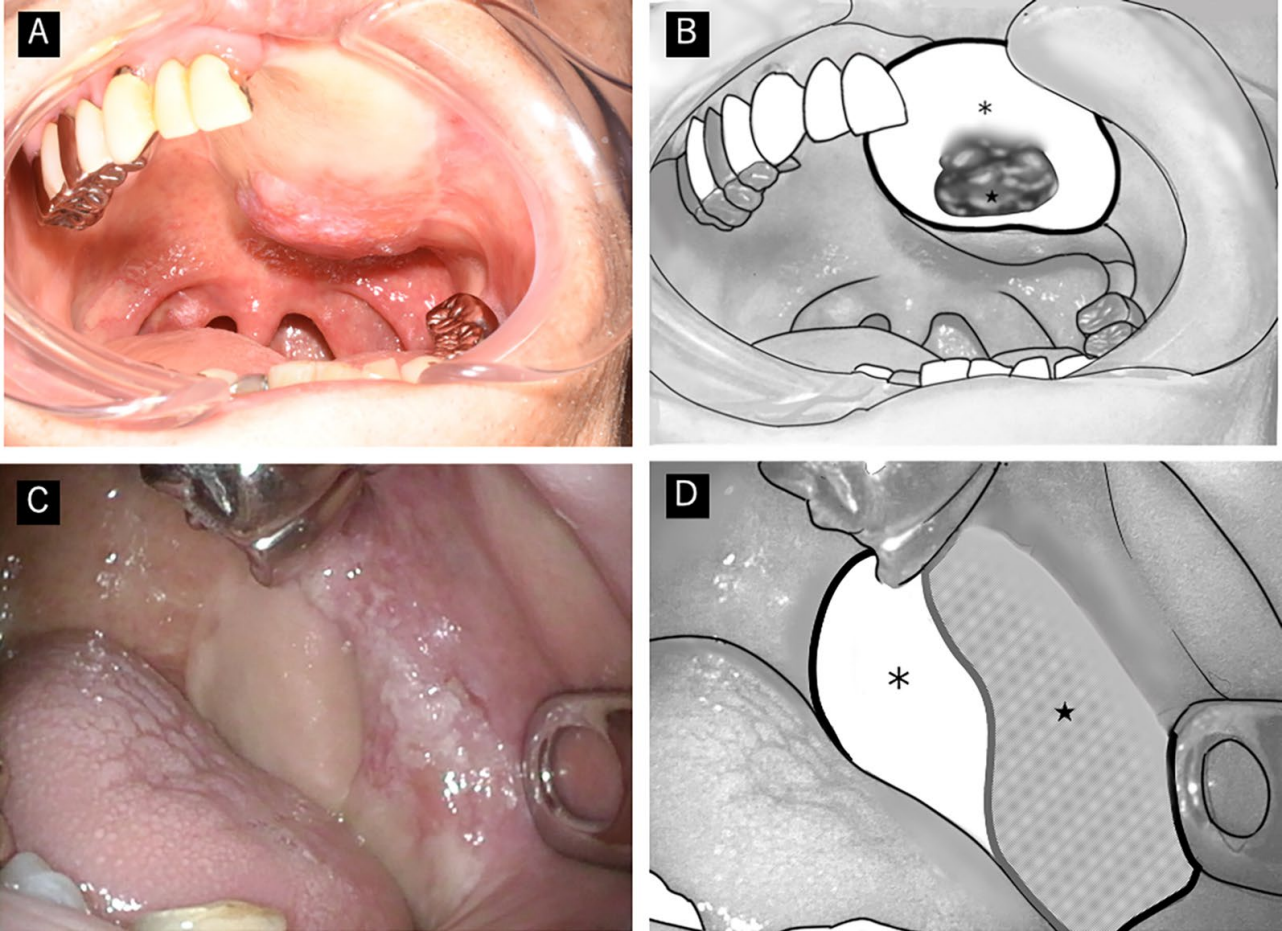
Gross findings and schematic representation of secondary neoplasms arising in the skin paddles of flaps. (**A**) Erythema with partial elevation in the center of the skin paddle. Biopsy revealed a well-differentiated squamous cell carcinoma. (**B**) Schematic representation of (**A**). This lesion showed typical macroscopic features of flap cancers. (**C**) Erythema with a partial elevation in the skin paddle, partially extending to the skin-mucosa junction. Biopsy revealed a well-differentiated squamous cell carcinoma. (**D**) Schematic representation of (**C**). This lesion was diagnosed as flap cancer in this study, although conventional classification would have resulted in a different diagnosis.

Characteristics of the patients are described in Table 1. Seven cases were defined as definite flap cancers, and all were squamous cell carcinomas. The mean latency interval was 114 months (range 24–312). The mean number of biopsies required to diagnose flap cancer was 2.0 (range 1–4) (Fig. 1). Lesions were located on the central and peripheral parts of the skin paddle in six and one cases, respectively (Fig. 2).

**Table 1:** Basic information of the SNAF cohort and characteristics.

Case	Age (yo)	Flap neoplasm type	Primary site	Flap	Adjuvant	Interval	Biopsy time	Flap cancer site	Macroscopic feature	IHC		Sequence	Therapy	Prognosis
					Therapy	(Months)				p53	p16	*TP53*		
1	64	Cancer	FOM	VFG	None	118	1	Center	Protruding	++	–	*	Local resection	10 years RFS
2	65	Cancer	Tongue	RFA	None	312	3	Center	Papillary	+ /−	+	NAD	Total flap resection	1 year DOD
3	71	Cancer	Tongue	ALT	RT	84	4	Center	Papillary	+ /−	+	NAD	Local resection	5 years RFS
4	69	Cancer	Tongue	ALT	None	180	3	Center	Protruding	+/−	–	NAD	Total flap resection	10 years RFS
5	54	Cancer	Tongue	ALT	None	36	1	Center	Protruding	–	+	NAD	Follow-up	1 year DOO
6	86	Cancer	Lower gum	RAMC	RT	43	1	Peripheral	Protruding	+/−	+	NAD	Local resection	5 years RFS
7	70	Cancer	Upper gum	RAMC	CRT	24	1	Center	Papillary	++	–	missense_ c.734G > T	Local resection	10 years RFS
8	68	Precancerous	Tongue	ALT	None	144	2	Center	Protruding	+/−	+	NAD	Follow-up	11 years RFS
9	74	Precancerous	Oropharynx	ALT	None	156	7	Center	Papillary	++	–	missense c.526 T > G	Follow-up	2 years RFS
10	84	Precancerous	Hypopharynx	RFA	None	92	5	Center	Papillary	–	–	stop gained_ c.438G > A	Follow-up	5 years RFS
11	69	Precancerous	Oropharynx	ALT	None	68	2	Center	Papillary	+/−	+	NAD	Follow-up	6 years RFS
12	68	Precancerous	Oropharynx	ALT	RT	46	1	Center	Protruding	+ /−	+	NAD	Follow-up	3 years RFS
13	68	Precancerous	FOM	NA	None	168	3	Center	Protruding	+/−	–	NAD	Local resection	8 years RFS
14	79	Precancerous	Tongue	RFA	None	108	3	Center	Protruding	+	+	missense c.480_481delGGinsAA	Follow-up	2 years RFS
15	78	Precancerous	Tongue	NA	None	312	1	Peripheral	Papillary	+	+	missense c.28G > A	Follow-up	6 years DOO
16	50	Precancerous	Tongue	ALT	None	2	1	Peripheral	Protruding	+/−	+	NAD	Follow-up	9 years RFS
17	56	Precancerous	FOM	ALT	None	17	2	Peripheral	Protruding	+/−	–	NAD	Follow-up	6 years RFS
18	82	Precancerous	Buccal	Local	None	83	3	Center	Papillary	+/−	+	NAD	Follow-up	7 years RFS
19	84	Precancerous	Buccal	Local	None	95	2	Center	Papillary	+/−	+	NAD	Follow-up	1 year RFS
20	70	Precancerous	Tongue	RAMC	None	72	2	Center	Protruding	+/−	–	NAD	Follow-up	2 years RFS
21	80	Precancerous	Buccal	ALT	None	150	3	Center	Protruding	+/−	–	NAD	Local resection	1 year RFS

SNAF, Second primary neoplasm arising in the skin paddle of the reconstructive flap; FOM, Floor of mouth; VFG, Vascularized fibular graft; RFA, Radial forearm flap; DP, Deltopectoral flap; ALT, Anterolateral thigh flap; RAMCF, Rectus abdominis myocutaneous flap; NA, Not available; RT, Radiotherapy; CRT, Chemoradiotherapy; RFS, Relapse-free survival; DOD, Died of disease; DOO, Died of other reasons.*Fail dut to poor DNA quality, NAD: No alteration detected.

**Figure 3:**
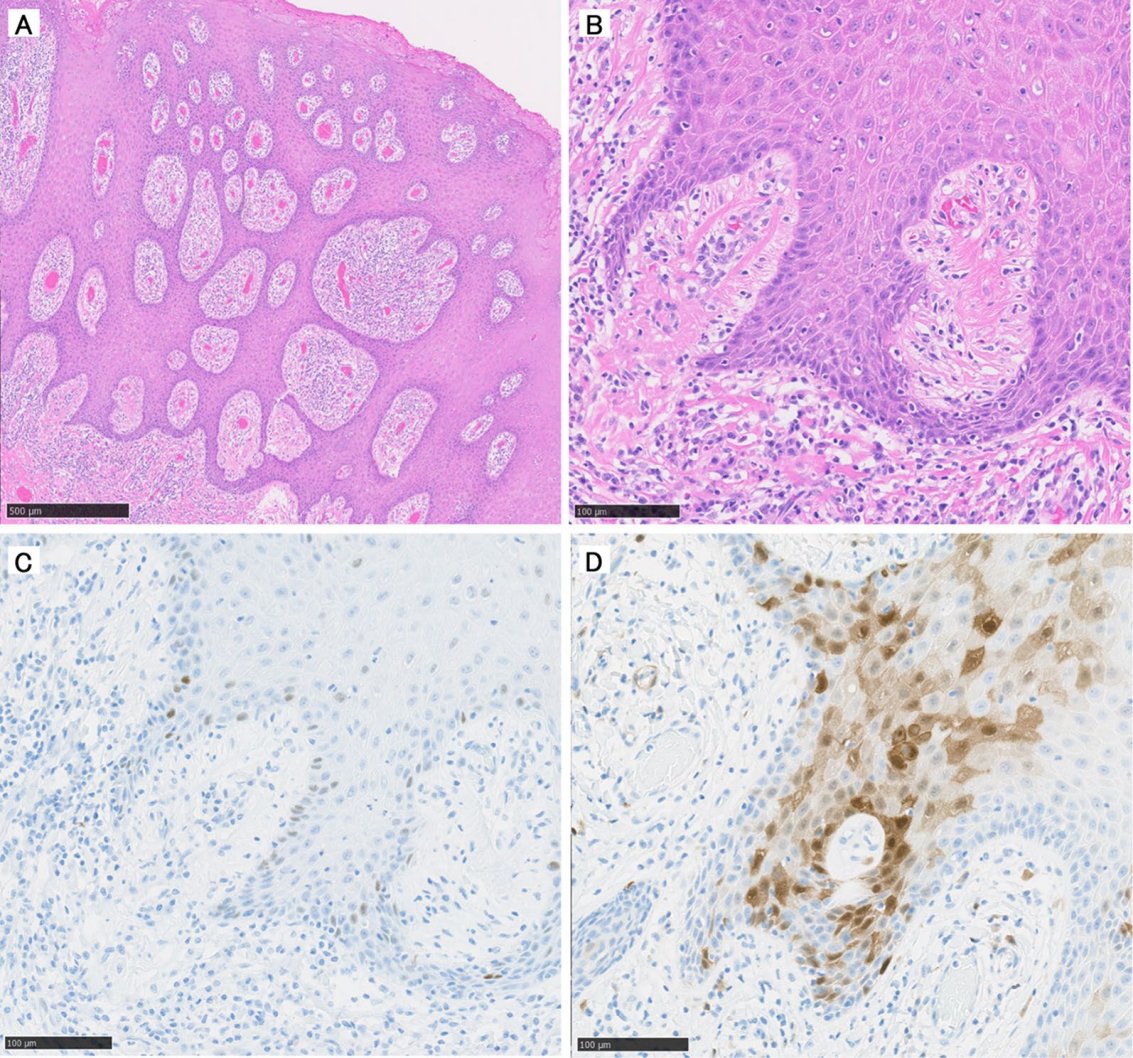
Histologic findings of flap cancer. Excessive keratinization was identified in the epithelium, accompanied by a strong inflammatory response in the submucosal layer. (**A**) Low power view. (**B**) High power view. (**C**) The staining result for p53 was + /− (wild-type). (**D**) The staining result for p16 was positive.

On macroscopic evaluation, all lesions protruded, and features of infiltrative growth, including ulcers, were negative. Furthermore, on histological evaluation, excessive keratinization was identified in the epithelium, accompanied by strong inflammatory features in the submucosal layer (Fig. 3). On IHC staining, 43% (three cases) showed altered p53 types, and 57% (four cases) were positive for p16. All but one lesion was eligible for NGS; mutation rate of *TP*53 was 17% (one case). Concordance rate of p53 and *TP*53 status was 83% (five cases); no *TP* 53 mutation was detected in one p53 altered-type case (Table 2).

**Table 2:** Comparison of baseline characteristics between the typical head and neck squamous cell carcinoma group and the two flap neoplasm groups.

	Control	Flap cancer	Precancerous lesion	*P*-value
	(*N* = 284)	(*N* = 7)	(*N* = 14)	
	No. of patients (%)			
Age at study entry, years				
> 75	219 (77%)	2 (29%)	6 (43%)	
< 75	65 (23%)	5 (71%)	8 (57%)	*p* < 0.001*
Sex				
Male	220 (77%)	5 (71%)	12 (86%)	
Female	64 (23%)	2 (29%)	2 (14%)	*p* = 0.69*
Tumor site				
Oral cavity	153 (54%)	7 (100%)	10 (71%)	
Hypopharynx	74 (26%)	0 (0%)	1 (7%)	
Oropharynx	32 (11%)	0 (0%)	3 (22%)	
Larynx	25 (9%)	0 (0%)	0 (0%)	*p* = 0.15*
p53 (IHC)				
Wild-type	65 (23%)	4 (57%)	10 (71%)	
Altered type	219 (77)	3 (43%)	4 (29%)	*p* < 0.001*
p16				
Positive	20 (7%)	4 (57%)	9 (64%)	
Negative	264 (93%)	3 (43%)	5 (36%)	*p* < 0.001*

Control: a historical cohort of patients with typical head and neck squamous cell carcinoma.

*Fisher’s exact test.

Therapies for flap cancers were as follows: total resection of the flap in two cases, local resection of the flap in four cases, and active surveillance in one case. Of the six cases that underwent resection of the lesions, the postoperative diagnosis was flap cancer in all cases. Lesions were carcinoma in situ in two cases. Regarding the remaining four cases, including the one fatal case, lesions were mainly limited to the epithelium with scant stromal invasion in very small areas. In all six cases, deep margins were clear, and horizontal margins were all positive for dysplasia or carcinoma in situ.

In one patient who was undergoing long-term immunosuppressive therapy with a history of systemic lupus erythematosus and interstitial pneumonia, flap cancer showed rapid progression. Despite radical resection, including total resection of the flap and adjacent structures such as the mandible and tongue with negative surgical margins, the patient died of the disease owing to uncontrolled rapid local relapse. This was the only patient who died of the disease in this study (Table 1).

### Precancerous lesions

Fourteen patients showed precancerous lesions. The mean latency interval was 108 months (range 2–312). In addition, biopsies were repeatedly performed for the same lesion with a mean number of 2.5 (range 1–7) repetitions (Fig. 1).

**Figure 4:**
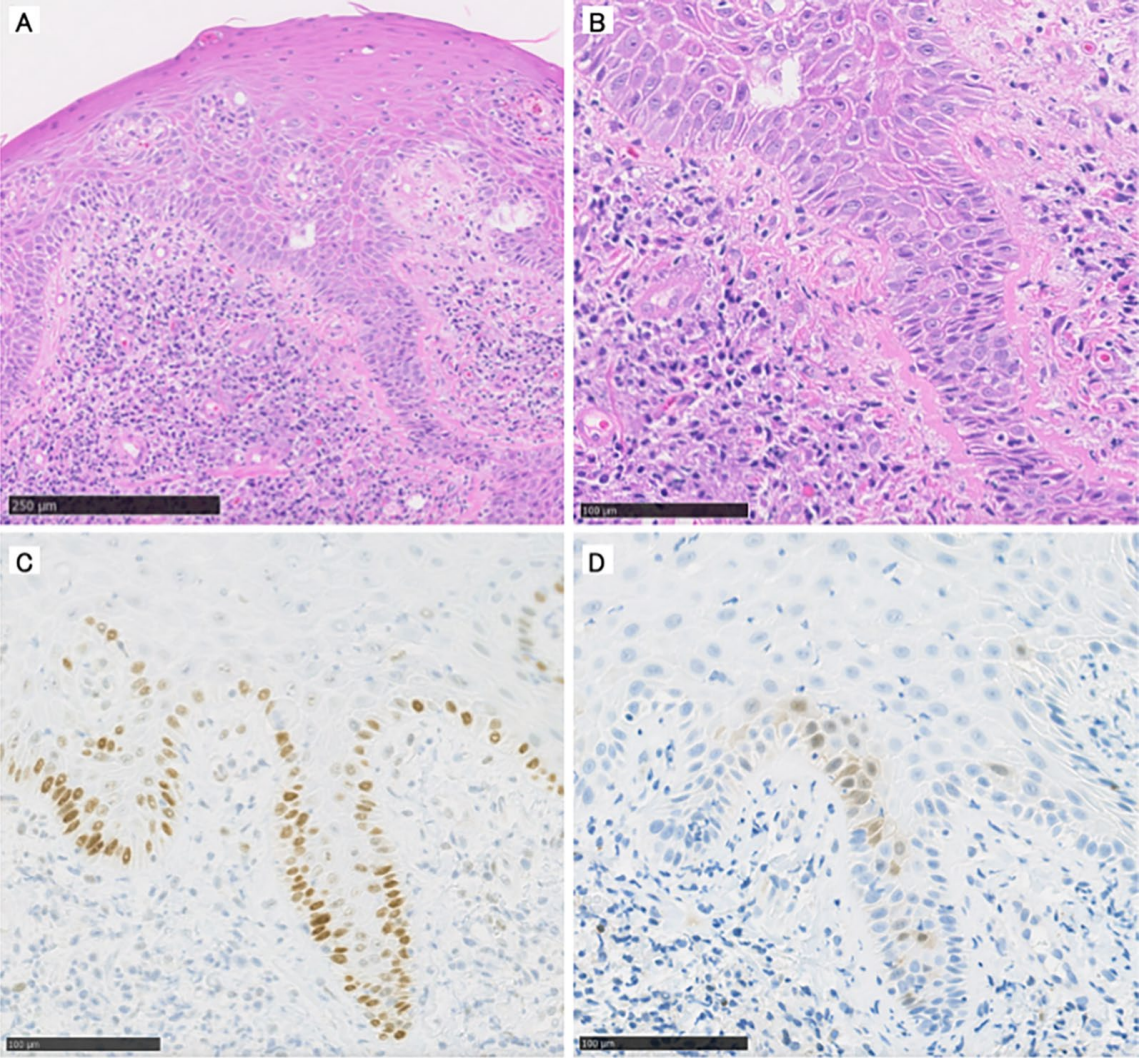
Histologic findings of precancerous lesions. Excessive keratinization was identified in the epithelium, accompanied by a strong inflammatory response in the submucosal layer. (**A**) Low power view. (**B**) High power view. (**C**) The staining result for p53 was +/− (wild-type). (**B**) The staining result for p16 was negative.

**Figure 5:**
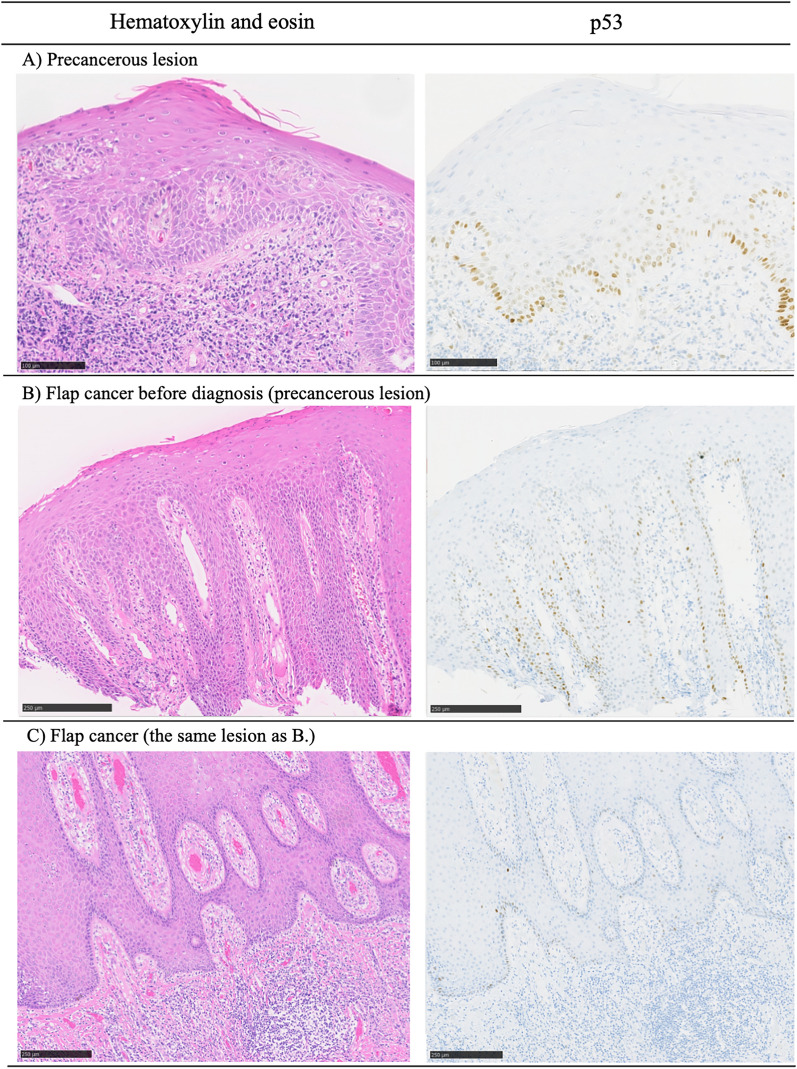
Sequential changes of second primary neoplasm arising in the skin paddle of the reconstructive flap. Histological transformation from a precancerous state to carcinoma of the flap over time is described. (**A**) shows a precancerous lesion that did not become flap cancer in the present study. The histological findings of the lesions in (**A**) and (**B**) are similar. The lesions in (**B**) and (**C**) are the same; however, (**B**) shows the lesion 2 years prior, showing the histological change over time.

On a macroscopic evaluation, all lesions protruded, and features of infiltrative growth, including ulcers, were negative. Moreover, on histological evaluation, excessive keratinization was identified in the epithelium, accompanied by strong inflammatory features in the submucosal layer (Fig. 4). On IHC staining, 7% (one case) showed altered p53 types, and 64% (nine cases) were positive for p16. These macroscopic and histological findings of precancerous lesions closely resembled those of the preceding lesions that later became flap cancers (Fig. 5). All lesions were eligible for NGS; mutation rate of *TP*53 was 28% (four case). Concordance rate of p53 and *TP*53 status was 100% (14 cases) (Tables 1 and 2).

Therapy for precancerous lesions was performed as follows: partial resection of the flap and active surveillance in one and 13 cases, respectively. None of the patients died of the disease (Table 1).

### Discrepancies between the SNAF group and the control cohort

The clinical and pathological characteristics of the three groups are presented in Table 2. At study entry, the control cohort was significantly older than the SNAF group (*p* < 0.01). The SNAF group showed a significant trend toward wild-type p53 and p16 positivity (*p* < 0.01). In addition, survival analysis revealed that the SNAF group was significantly associated with better survival than the control cohort (*p* = 0.04) (Supplementary Material 3).

## Discussion

In this study, we presented 21 SNAFs, comprising seven cases of flap cancer and 14 cases of precancerous lesions from a single center over 20 years. The findings of this study indicate that the typical features of SNAFs are as follows: (1) they are grossly exophytic with highly inflamed stroma, (2) they are difficult to diagnose and require repeated biopsies, (3) they tend to show a relatively slow-growing feature, and (4) when compared with ordinary HNSCC, SNAFs exhibit a lower incidence of altered p53 type/TP53 mutation and a relatively high p16 positivity rate^22^. The correlation between p53 phenotype and *TP*53 mutation was considered reasonable given the sensitivity and specificity^22^. In addition, clinicopathological features, diagnostic measures, optimal treatment, and possible mechanisms of carcinogenesis are discussed.

This study implies that SNAF is essentially an exceptionally highly differentiated squamous cell carcinoma; flap cancers and precancerous lesions merely represent different stages of the same phenomenon. Indeed, in this study, physicians tended to repeatedly biopsy the same lesions before obtaining a final diagnosis (Fig. 1). Based on this study’s cohort and previous literature^16^, SNAF is assumed to be a slow-growing tumor with a good prognosis. Although we experienced one exceptional case with a poor prognosis, we speculate that the patient’s history of autoimmune disease and the influence of long-term immunosuppressive therapy may have negatively altered the pathophysiology. It may be beneficial for physicians to be aware of this unique second primary neoplasm as it is expected to be curable if carefully monitored and treated; excessive surgery may not be necessary in most cases.

A consensus on the diagnostic criteria of SNAF does not exist; hence, past studies conventionally defined lesions fulfilling the following five features as flap cancers: (1) there is no tumor remnant after the initial treatment; (2) some years have elapsed since the end of initial treatment; (3) there are no skin malignancies elsewhere on the body; (4) the tumor is limited to the skin flap and is far from the oral mucosa; and (5) skin squamous cell carcinoma is confirmed by histopathological examination^10,11,16^. However, the authors believe that the above criteria need not be fulfilled universally. For example, even if the initial surgical margins were positive, if the lesion originated from the center of the skin paddle, judging the lesion to be a local recurrence would be spatially inconsistent; such a lesion should also be diagnosed as SNAF. In addition, even if skin cancer develops simultaneously or atypically throughout the body, it seems reasonable to consider this as an independent lesion if there is a physical distance from the donor site. Furthermore, if a neoplasm arising from the skin paddle advances, it will eventually invade the surrounding mucosa, and these lesions should be diagnosed as SNAF. Finally, as this study suggests that flap cancer is an exceptionally highly differentiated squamous cell carcinoma and does not differ significantly from precancerous lesions, we recommend that the two should be broadly diagnosed as SNAF. The latter should not be excluded from the disease concept. Therefore, new diagnostic criteria based on carcinogenic mechanisms are desirable while referring to conventional measures.

There is no consensus regarding the carcinogenic mechanisms of SNAFs. Although the effects of alcohol consumption, smoking, postoperative radiotherapy, and human papillomavirus infection have been discussed in the literature^25,26^, a strong correlation has not been demonstrated for these factors. A reasonable explanation might be that neoplastic keratinocytes derived from the intraepithelial oral and pharyngeal mucosa spread into the skin paddle and produce new cancer, a theory proposed and described by Braakhuis et al.^27^ as the “patch-field carcinoma model.” However, in this hypothesis, the new neoplasm in the skin paddle would be an ordinary HNSCC; therefore, IHC staining should show a high rate of altered p53 type and negative p16 status.

We believe that carcinogenesis induced by chronic irritation, frequent infection, repeated trauma, and contraction of the alien oral-pharyngeal environment to the grafted skin is the most likely cause; in other words, SNAFs are basically skin cancers. Reconstructive flap skin has been reported to develop severe epithelial dysplasia with concomitant deregulation of proliferation and increased p53 expression^28^. Additionally, carcinogenesis caused by chronic irritation is a well-known etiology, and a similar concept includes burn scar carcinogenesis^29–31^. Previously, “mucosalization,” the loss of skin appendages including sebaceous glands in the transplanted skin paddles, has been noted in the literature and is considered an inflammatory change caused by chronic irritation^32,33^. Therefore, we believe that SNAF is also a product of inflammatory changes and is very similar to burn carcinomas^29,30^. The problematic difference between the two is that although burn scar carcinoma is expected to decrease in the coming years owing to the refinement of burn scar therapy^30^, one would expect to see more cases of SNAF as the number of flap reconstructions is increasing and patients with cancer are living longer.

The significance of this study is limited for two reasons. First, the number of SNAFs detected was small; nevertheless, this study had a relatively large cohort than previous reports. Second, the method used to explore the etiology of SNAFs has limitations. To further evaluate the etiology of SNAF, the search range and methods may need to be expanded. Although we evaluated the p53 phenotype along with *TP*53 status with IHC and NGS, our sequence was targeted to frequently mutated regions of *TP*53. Broader evaluation, such as whole exome sequence and other techniques that were not employed in this study may be considered to explain the etiology of the high p16 positive rate of SNAF and the accurate distinction between the recurrence of the original disease and SNAF.

In conclusion, patients undergoing long-standing flap reconstruction surgery may benefit from careful monitoring for SNAF. SNAFs are grossly exophytic, pathologically accompanied by highly inflamed stroma, and exhibit relatively benign behavior. Pathological atypia is not conspicuous, but fairly frequent incidence of altered p53 type/*TP*53 mutation and a higher p16 positivity rate than ordinary HNSCC is detected. Diagnosis is often difficult; therefore, it may advisable to perform repeated or excisional biopsy of the lesion when SNAF is suspected.

## Supplementary Information

Supplementary Information.

## Data Availability

The datasets generated and/or analysed during the current study are available in the NBDC human data repository, [Now registering].
