# Supplementary material for: Clinical and pathological features of second primary neoplasms arising in head and neck reconstructive skin flaps
Source: Sci Rep. 2023 Jul 11;13:11214. doi: 10.1038/s41598-023-38122-9 (PMC10336017; doi:10.1038/s41598-023-38122-9)
Supplement: Supplementary file 1 — Supplementary Information. [file 41598_2023_38122_MOESM1_ESM.pdf]

Clinical and pathological features of second primary neoplasms arising in head and neck reconstructive skin flaps

Kohtaro Eguchi[a], Kenya Kobayashi[a,b], Yoshitaka Honma[c], Eijitsu Ryo[c, f], Airi Sakyo[d], Kazuki Yokoyama[c], Takane Watanabe[a], Yusuke Aihara[a], Azusa Sakai[a], Yoshifumi Matsumoto[a], Toshihiko Sakai[a], Go Omura[a], Yasushi Yatabe [d, f], Seiichi Yoshimoto[a], Taisuke Mori[*d,]

[a]Department of Head and Neck Surgery, National Cancer Center Hospital, 104-0045, 5-1-1, Chuo-ku, Tokyo, Japan

[b] Department of Otolaryngology, Head and Neck Surgery, The University of Tokyo, 113-8655 7-3-1, Hongo, Bunkyo-ku, Tokyo, Japan

[c]Department of Head and Neck, Esophageal Medical Oncology, National Cancer Center Hospital, 104-0045, 5-1-1, Chuo-ku, Tokyo, Japan

[d]Department of Diagnostic Pathology, National Cancer Center Hospital, 104-0045, 5-1-1, Chuo-ku, Tokyo, Japan

[f]Division of Molecular Pathology, National Cancer Center Research Institute, 104-0045, 5-1-1, Chuo-ku, Tokyo, Japan

Correspondence to: Taisuke Mori

Supplementary Material 1: Reported flap cancer cases

Case	Author, year	Age (years)	Sex	Subsite	Flap	Interval (years)	Macroscopic findings	Pathology	Therapy
1	Yoshino, 1989 [2]	74	F	Hypopharynx	DP	8	Protruding	SCC	Local resection
2	Yoshino, 1989 [2]	78	F	Hypopharynx	Local	6	NA*	SCC	NA*
3	Yoshino, 1989 [2]	76	F	Hypopharynx	DP	16	NA*	SCC	NA*
4	Deans, 1990 [3]	61	M	Larynx	DP	24	Protruding	SCC	Total flap resection
5	Scott, 1992 [4]	67	M	Floor of mouth	Acromio-thoracic tube pedicles	43	Protruding	SCC	Local resection
6	Sa'do, 1994[5]	70	F	Lower gum	ALT	25	Protruding	SCC	Local resection
7	Sakamoto, 1998 [6]	63	M	Hypopharynx	RFA	10	Scattered	SCC	Total flap resection
8	Ohtsuka, 1998 [7]	72	F	Lower gum	PMMC	8	Protruding	SCC	Local resection
9	Iseli, 2002 [8]	67	M	Larynx	DP	27	Protruding	SCC	Total flap resection
10	Monnier, 2008 [9]	62	M	Oropharynx	RFA	3.5	Protruding	SCC	Local resection
11	Monnier, 2008 [9]	54	M	Oropharynx	DP	20	Thickening	SCC	Local resection
12	Ho, 2011 [10]	65	M	Tongue	PMMC	12	Indwelling	SCC	Total flap resection
13	Zemann, 2011 [11]	62	F	Hard palate	Jump skin flap	30	Protruding	SCC	Total flap resection
14	Yamasaki, 2011 [12]	75	M	Hypopharynx	RFA	5	Protruding	SCC	Total flap resection
15	Foschini, 2011 [13]	61	F	Lower gum	RFA	4	Protruding	SCC	Local resection
16	Foschini, 2011 [13]	58	F	FOM	RFA	4	Indwelling	SCC	Local resection
17	Foschini, 2011 [13]	52	M	Tongue	RFA	5.5	Protruding	SCC	Local resection
18	Nasu, 2012 [14]	75	M	Tongue	RFA	16	Exophytic	SCC	Total flap resection
19	Tokita, 2013 [15]	80	M	Hypopharynx	RFA	5.5	Erythema	SCC	Local resection
20	Cymerman, 2013 [16]	62	M	Floor of mouth	RFA	23	Exophytic	SCC	Local resection
21	Valentini, 2016 [17]	72	F	Tongue	RFA	21	Leukoplakia	SCC	Total flap resection
22	Terauchi, 2019 [18]	67	F	Buccal	ALT	5	Exophytic	SCC	Local resection
23	Amin, 2019 [19]	62	M	Hypopharynx	PMMC	2	Exophytic	SCC	Local resection
24	Nakaue 2021 [20]	75	M	Tongue	PMMC	21	Exophytic	SCC	Local resection

* NA: not available; no details were provided in this paper. F, female; M, male; FOM, floor of mouth; DP, deltopectoral flap; PMMC, pectoralis major myocutaneous flap; ALT, anterolateral thigh flap; RFA, radial forearm flap; SCC, squamous cell carcinoma

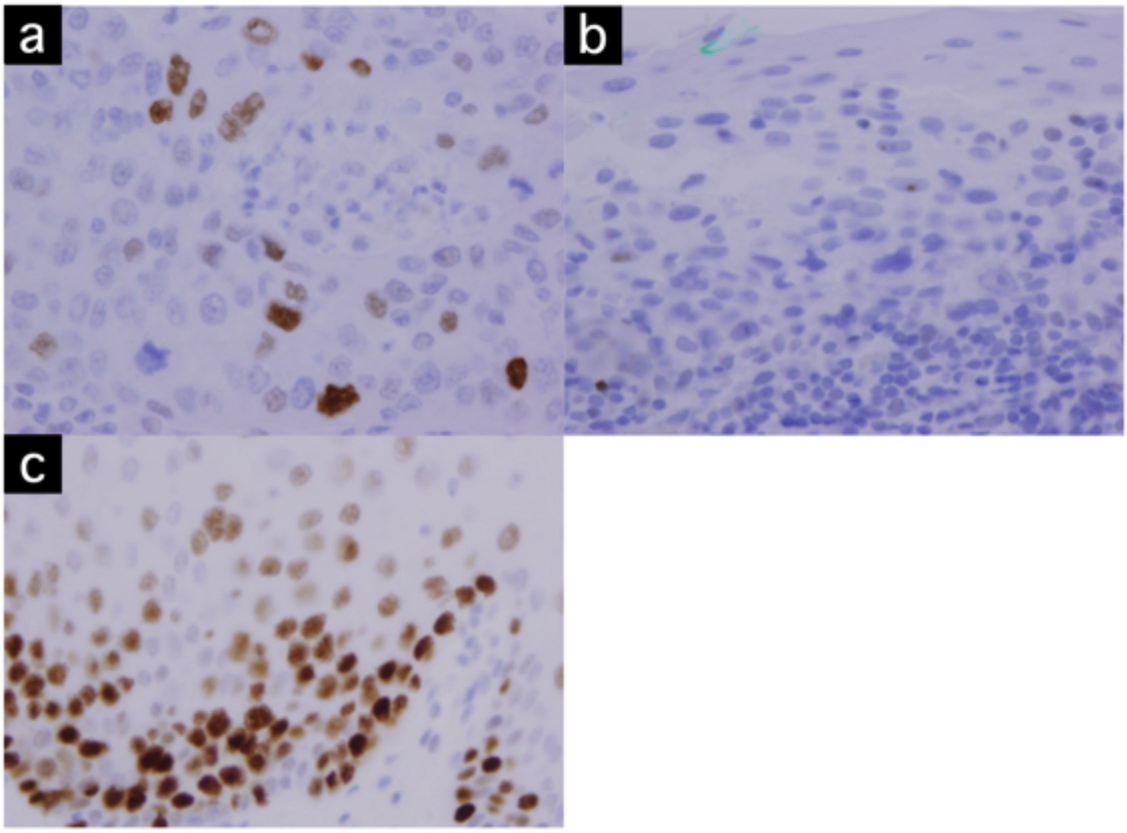

Supplementary Material 2: p53 immunohistochemical staining patterns

a) +/- : Non-regionalized patchy stains.

b) - : Lacking any stain (lost).

c) ++ : Strong regionalized stains (accumulation).

Stain patterns of +/- was judged as wild type.

Stain patterns of - and ++ were judged as altered type.

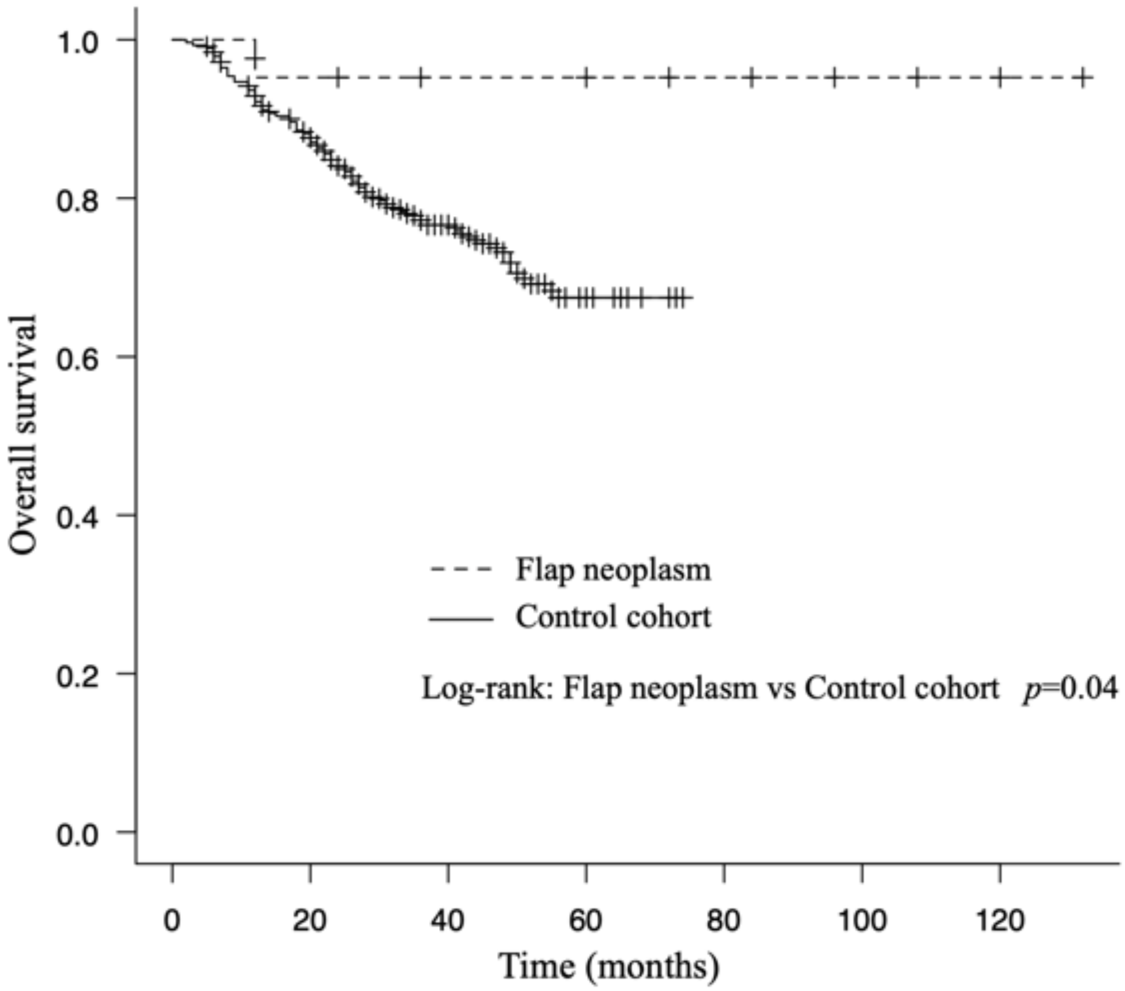

Supplementary Material 3: Unadjusted Kaplan–Meier disease-specific survival curves
Survival time was defined as the number of survival months from the date of the last biopsy and the completion of initial therapy for the flap neoplasm group and the control cohort, respectively, until the date of death or the last visit. The flap neoplasm group was associated with significantly better disease-specific survival (log-rank: $p = 0.04$).
